# The efficacy and safety study of dietary supplement PURIAM110 on non-insulin taking Korean adults in the stage of pre-diabetes and diabetes mellitus: protocol for a randomized, double-blind, placebo-controlled, and multicenter trial-pilot study

**DOI:** 10.1186/1745-6215-12-38

**Published:** 2011-02-11

**Authors:** Sunju Park, Jeong-Su Park, Hoyeon Go, Bo-Hyoung Jang, Yongcheol Shin, Seong-Gyu Ko

**Affiliations:** 1 Center for Clinical Research and Genomics, College of Oriental Medicine and Institute of Oriental Medicine, Kyung Hee University, 1 Hoegi-dong, Seoul, Republic of Korea; 2 Department of Internal Medicine, College of Oriental Medicine, Semyung University, Bongbang-dong, Chungbuk, Republic of Korea; 3 Health Technology Assessment Research Division, National Evidence-based Healthcare Collaborating Agency, Wonnam-dong, Seoul, Republic of Korea

## Abstract

**Background:**

Diabetes has already become a threat to the nation and the individual due to its high prevalence rates and high medical expenses. Therefore, preventing diabetes at an earlier stage is very important. Despite advances in antidiabetic agents, we have not yet achieved any satisfying results in treating diabetes. Among various treatments, medicinal herbs and supplements for diabetes are reported to show generally good efficacy and safety data. In particular, PURIAM110, a compound from orange fruits and mulberry leaves, is supposed to prevent the progress of type II diabetes mellitus and improve diabetic symptoms. This is the first reported pilot study about the protective effect of the orange fruits and mulberry leaves mixture against pre-diabetes on Korean adults. Based on these positive results of herb-derived components, extended studies of dietary supplements have to be done to suggest confirmative evidences.

**Methods/Design:**

The efficacy and safety study of PURIAM110 is a double-blinded, placebo-controlled, randomized, and multi-center clinical trial. A total of 45 subjects will participate in this study for 6 weeks.

**Discussion:**

The present protocol will confirm the efficacy and safety of PURIAM110 for pre-diabetes, suggesting more basic knowledge to conduct further randomized controlled trials (RCT). In addition, PURIAM110 can be an alternative dietary supplemental remedy for diabetes patients.

**Trial Registration:**

ISRCTN: ISRCTN44779824

## Background

Pre-diabetes is a condition of potential diabetes mellitus with an increased risk of developing type 2 diabetes [1,2]. In other words, it is a state of either impaired fasting glucose (IFG) or impaired glucose tolerance (IGT). Pre-diabetes is asymptomatic in many occasions, but once it develops into type 2 diabetes, it makes patients suffer from polydipsia, polyuria, polyphasia, unusual weight loss, and extreme fatigue [3]. Unmanaged chronic diabetes mellitus affects quality of life and induces life-threatening diabetes-associated complications [4,5]. Its mortality rate cannot be ignored in Korea, becoming the fourth leading cause of death by 2030 [6]. According to the Korean National Health and Nutrition Survey (KNHNS) 2001, prevalence rates of diabetes were 1.4 million (8.1%) in Korean men and 1.3 million (7.5%) in Korean women. Nowadays, it is reported that about 308 million people worldwide are having impaired glucose tolerance (IGT) and among them 25% to 75% will develop diabetes [7,8]. Since the exact figures of Korean pre-diabetes patients and diabetes progressing rates were not reported [4], we can deduce from the above numerical statement that a considerable population *must be *at the status of potential diabetes. Socioeconomic expenditures are also high, at 193 million won per year for medication [4,9]. Therefore, preventing and treating efforts for the non-insulin taking patients in the stage of pre-diabetes and diabetes are urgently needed to lift the burden from both the nation and patients [10]. Even though novel remedies have been produced unceasingly, most of them seem to be often ineffective for this disease [11,12]. Meanwhile, dietary supplements are known to be relatively effective in treating diabetes, with few adverse effects [13-15]. Among 400 reported herbal medicines for diabetes [16], recent studies suggests that bitter oranges and mulberry leaves have antidiabetic effects. PURIAM110, a compound made from orange fruits and mulberry leaves (patent application no. 10-2006-0020040), is expected to prevent the progress to the early stage of diabetes mellitus, safely [17,18]. Bitter orange is a dried, immature fruit of *Citrus aurantium L*., used from ancient times to remove the stagnation and distension in hypochondriac regions [19,20]. Like other herbal components, its mechanism of action seems to be complex though, the fruit has been identified to produce antihyperglycemic activity. Mulberry leaf is a dried leaf of *Morus indica L*., which also shows hypoglycemic properties [21,22]. It has been prescribed widely to dispel wind-heat and heat in the lung [23,24]. Relying on pathophysiologic mechanisms from traditional Korean medicine, diabetes can be caused when there is heat in the lung or a deficiency of energy (Yang qi) and body fluid. Therefore, bitter orange's qi driving activity and mulberry leaf's heat dispelling function in the lung can be applied to play an antidiabetic role.

Despite the advantages of herb-derived supplements, few RCTs have been conducted [8,25], and the bitter oranges and mulberry leaves compound is not an exception. Further trials of dietary supplements have to be done to suggest confirmative evidence.

The aim of this study is to evaluate the efficacy and safety of the PURIAM110, a dietary supplement for the treatment of patients with pre-diabetes and diabetes mellitus non-insulin dependent stage [26]. This paper is the first pilot study with orange fruits and mulberry leaves on Korean adults who are at the status of pre-diabetes and diabetes mellitus not in the insulin requiring stage. The results of this study will give us the clinical of biochemical parameters, such HbA1c, fructosamine, fasting glucose and 2-h OGTT glucose between the placebo and PURIAM110 groups. On the basis of the results, we can suggest the optimal design, precise sample size, and primary outcome for a further large scale RCT.

## Methods/design

### Objectives & Hypothesis

#### Objectives

The primary objective of this pilot study is to assess the efficacy and safety of PURIAM110 on non-insulin taking Korean adults in the stage of pre-diabetes and diabetes mellitus.

The secondary objective is to estimate the precise sample size and primary endpoint required for the large scale RCT.

#### Hypothesis

We hypothesize that glucose concentration of pre-diabetes and diabetes mellitus who are treated with PURIAM110 will be lower than that of the control group. Also, we expect that other diabetes related parameters (total cholesterol, triglyceride, and LDL cholesterol) to be improved and the symptoms to be normalized in the treatment group.

### Study design

This is a 6-week, double-blinded, placebo-controlled, randomized, and multi-center clinical trial assessing the efficacy and safety of PURIAM110.

The study has been conducted in two Oriental Medical Hospitals in Korea from December 2006.

**Figure 1:**
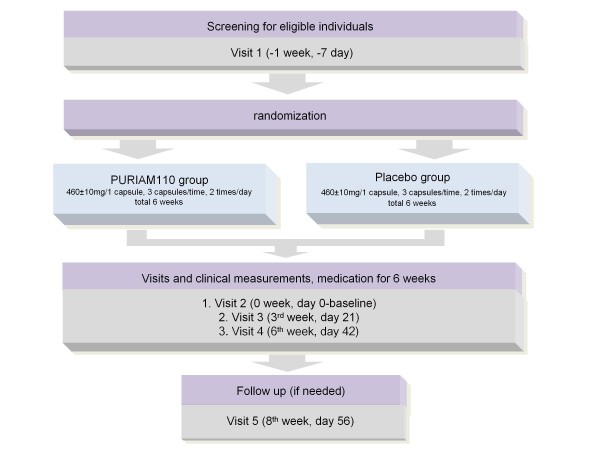
**Study flowchart**. Summary of the study flow.

Figure 1 provides an overview of the study.

### Population

The Korea Food & Drug Administration (KFDA)'s rule recommends including not only healthy group but also a sub-health condition population when conducting dietary supplement trials. Therefore, we included pre-diabetes status, and diabetes mellitus non-insulin dependent stage Korean adults, aged 18 to 65. The detail inclusion and exclusion criteria are described in Table 1 and Table 2.

**Table 1:** Inclusion criteria. Inclusion criteria of the PURIAM110 trial

Inclusion criteria	
1.	Both gender between the ages of 18-69 years, eligible for study through screening test prior to the enrolment.
2.	Able and willing to perform the study protocol and participate throughout the entire trial period(Screening, baseline, 3, 6 weeks, follow up(if needed)).
3.	Participants who understood the risks and benefits of the study and signed a written informed consent.
4.	Among individuals not under diabetes mellitus treatment, whose random glucose concentration is 110~250 mg/dL measured with Accu-Chek^®^(Roche, Germany) glucometer within 3 weeks prior to participation.
4.1.	fasting plasma glucose concentration is 100-190 mg/dL or
4.2.	2- hour plasma glucose concentration* is 130-250 mg/dL( * venous plasma glucose 2-hour after ingestion of 75 g oral glucose load; DIASOL-S LIQ. [Glucose] Taejoon Pharm.)

**Table 2:** Exclusion criteria. Exclusion criteria of the PURIAM110 trial

Exclusion criteria	
1.	Presently using other blood glucose level controlling agents.
2.	History of allergy to the herbal products (bitter oranges and Mulberry leaves) or allergic diseases such as asthma.
3.	Medication (within the last 1 month or during the study) which would affect the study results.
4.	Daily intake of alcoholic beverages.
5.	Smokers consuming more than 1 pack/day.
6.	Presently having acute diseases or other untreated illness requiring treatment.
7.	Impaired hepatic or renal functions.
8.	Pregnant, breast feeding status or female of reproductive age, not using proper contraception.
9.	Participant in other clinical trials or a blood donor, within the past 1 month.
10.	History of severe disease or any condition, in the investigator's opinion, that would endanger the individual's safety or affect the study result.
11.	Patients diagnosed as type I and type II diabetes mellitus(insulin requiring stage).

### Inclusion and exclusion criteria

#### Inclusion criteria

Inclusion criteria are as follows (Table 1).

#### Exclusion criteria

Exclusion criteria of PURIAM110 trial are summarized in Table 2.

#### Suspension criteria

• Administration of forbidden medicine.

• Subject's demand to discontinue the study.

• Serious adverse events (AE) or unusual changes in clinical test results.

• Principal investigator's decision to terminate the study (low rates of compliance, complications, or unable to sustain the study for various reasons).

### Sample size

We had difficulties in determining adequate sample size, as preliminary studies were insufficient. So we adopted a pilot study design.

Two points had to be considered in deciding the sample size. One was the special condition of herb-derived dietary supplements regulatory law in Korea, and another was limited research fund. Since herbs have historical efficacy and safety profile in Korea, KFDA regulates dietary supplements under a different rule, compared to conventional drugs. In other words, as the efficacy and safety of herbal products have been clinically validated for a long time, the regulatory authorities have lightened restrictions, such as minimizing sample size. We have planned the dietary supplement study according to this KFDA rule. The KFDA rule prescribes that when designing a dietary supplements trial, the sample size has to be a minimum 30 per group. Therefore we first fixed the number of the subjects in the treatment group as 30.

As the research funding was restricted, we plan to allocate 15 individuals for the placebo group. When sample size is inevitably restricted, it is considered to be more ethical by the KFDA to set more subjects in the test group than in the control group. In conclusion, a total of 45 subjects will be recruited in this pilot study.

### Randomization

The study subjects will be randomly assigned to either PURIAM110 or placebo group at a ratio of 2:1, according to the 'random number table' generated by SAS software package (SAS institute, Inc, Carey, NC, USA, version 8.2).

### Blinding

The study drugs are double blinded to both investigator and subject. The contract research organization (CRO; Kyung Hee University, Center for Clinical Research and Genomics) of the sponsor, labels the investigational drugs by the randomization code number. The labeled experimental products will be provided to the trial sites by the CRO.

### Interventions

#### Treatment drug

PURIAM110 is a 460 mg hard gelatin capsule, containing dried powder of bitter oranges and mulberry leaves (Table 3). The dried powder is made of hot water extraction of bitter oranges and mulberry leaves 1:1 mixture followed by spray drying. The raw material extract is provided by SunTen pharmaceutical company (SUN TEN PHARMACEUTICAL Co., LTD.) in Taiwan.

**Table 3:** Constituents of PURIAM110. Constituents of the PURIAM110

PURIAM110 (460 mg/1 hard gelatin capsule)
bitter oranges (zhi shi, *Fructus Aurantii*; the unriped fruits of *Citrus aurantium L*.)
mulberry leaves (sang ye, the dried leaf of *Folium Mori*)
Sodium Silica Aluminate (lubricants)

#### Constituents of PURIAM110

The constituents of PURIAM110 are in Table 3.

#### Placebo

The placebo will contain the lactose. And the placebo will be manufactured to have a similar appearance, shape, weight (460 ± 10 mg/1 capsule), taste, and color with PURIAM110 [27].

#### Directions

The subjects will be prescribed for total 6 weeks, 2 times per day (3 capsules before breakfast and 3 capsules before dinner). Participants will take total 6 capsules daily, with a dose of 2,760 mg (460 ± 10 mg/1 capsule × 6 capsules).

### Study groups

1. Treatment group: PURIAM110.

2. Control group: placebo.

### Study period

The study period will be of 42 days (total 6 weeks). The visits and the evaluations will be done at the screening (visit1), 0th day (visit2; baseline), 21th day (visit3; 3rd week), and 42nd day (visit4; 6th week). 56th day is a follow up (if needed).

### Outcomes

#### Efficacy assessment index

##### Primary outcome index

1. Biochemical parameters:

1.1. HbA1c

1.2. Fructosamine

1.3. Fasting glucose

1.4. 2-h OGTT glucose

1.5. Fasting insulin

1.6. Total cholesterol

1.7. Triglyceride

1.8. Low density lipoprotein (LDL) cholesterol

##### Secondary outcome index

1. Diabetes symptoms*

1.1. polydipsia

1.2. polyuria(frequent urination)

1.3. polyphasia

1.4. fatigue

2. Other clinically significant parameters**

3. Anthropometric parameters:

3.1. body weight(kg)***

3.2. waist circumference(WC, cm)

3.3. hip circumference(HC, cm)

The primary and secondary outcomes will be measured at the 0th day (visit2; baseline), the 21th day (visit3; 3rd week), and the 42nd day (visit4; 6th week) during the treating period. And, when necessary, the outcomes will be assessed at the 56th day (follow-up). The changes between the 0th day (visit2; baseline) and the 42nd day (visit4; 6th week) measurements will be analyzed for the primary outcome indexes. For the secondary outcomes, a visual analogue scale (VAS) will be used to detect the improvement of diabetic symptoms (*). When the scale has improved more than two grades or a patient has recovered completely from these symptoms, it will be evaluated as 'effective'. For other categories (**), we will assess whether these parameters have normalized or not. Measured weight minus 0.5 kg (participant's garment weight) will be recorded as the subject's true weight (***).

#### Safety assessment index

All informations including vital signs, general medical examinations, laboratory test results, and adverse events will be recorded for the safety assessment.

### Procedures

#### Recruitment

Subjects will be recruited through two routes. Patients who visit the trial hospitals and meet the criteria will be recommended by a physician in charge. Those who see the trial poster on bulletin boards will visit the trial site voluntarily.

#### Study schedule & test items per visit

The detailed items which will be measured at every visit are described in Table 4.

**Table 4:** Study schedule. A brief study schedule at every visit

	Screening* Visit 1	Baseline Visit 2	Visit 3	Visit 4	Follow up (Visit 5)
	
	D-21 ~D-1	D0 Week 0	D21 Week 3	D42 Week 6	D56 Week 8
Informed consent form	●				

Demographic information taking ^1^	●				

Medical history taking	●				

Finger-prick blood glucose testing	●				

QSCC II ^2^	●				

Inclusion/exclusion criteria check	●				

Physical examination ^3^	●	●	●	●	

Vital sign measurement ^4^	●	●	●	●	

Laboratory test ^5^	●	●	●	●	

VAS ^6^	●	●	●	●	

Electrocardiogram(ECG)	●			●	

Concomitant drugs check	●	●	●	●	

Treating physician examination ^7^	●	●	●	●	

Adverse event monitoring			●	●	●

Compliance checking			●		

Study drug distribution		●	●		

Smoking, drinking, coffee taking status ^8^	●				

Diet, physical exercise Counseling ^8^		●	●		

●: Item need to be carried out during the visit.

* required to fast from 10 p.m. the day before the test prior to the blood sample collection.

^1 ^sex, date of birth, age, contact address and phone number.

^2 ^Questionnaire for the Sasang Constitution Classification II

^3 ^body weight(kg), height(cm), waist circumference(cm), hip circumference(cm).

^4 ^blood pressure(mmHg), pulse(/min), body temperature(°C).

^5 ^blood test (WBC, RBC, hemoglobin(HGB), hematocrit(HCT), platelet count(PLT), AST, ALT, γ-GTP, total bilirubin, albumin, total protein, total cholesterol, triglyceride, HDL cholesterol, BUN, creatinine, glucose(fasting), 2h-OGTT glucose, HbA1c, fructosamine, insulin(fasting)), urine test.

^6 ^visual analogue scale: polydipsia, polyuria, polyphasia, fatigue.

^7 ^includes present illness, past history taking.

^8 ^risk factors such as smoking, alcohol drinking, severe exercise and other drug intake will be managed strictly.

### Measurement tools

#### Visual analogue scale (VAS)

A 10 cm VAS (ranging from 0 cm as no symptom to 10 cm as the maximum symptom) will be used to assess diabetic symptoms (polydipsia, polyuria, polyphasia, and fatigue) improvement during treatment term.

#### Anthropometric measurements

The items will be measured with a standard operating procedure (SOP) by the well-trained physicians. All participants will be measured in light garments (estimated approximately 0.5 kg) and bare feet. The waist circumference (WC: recorded to the nearest 0.1 cm) will be taken 2.5 cm above the umbilicus at the upright position. The hip circumference (HC: to the nearest 0.1 cm) will be measured at the horizontal level of the widest part of the hip.

#### Questionnaire for the Sasang Constitution Classification II (QSCC II)

A QSCC II is a questionnaire which categorizes a person into four types of constitution by its characteristics, in traditional Korean medicine. We will use this questionnaire to classify Korean pre-diabetes and diabetes (not insulin requiring stage) patients. Also we will apply this questionnaire to evaluate the association between the pre-diabetes susceptibility and the patient's constitution. Moreover, we will use this to assess which constitution responds well to PURIAM110 [28].

### Compliance calculation

The researchers should provide the investigational drug to the randomized participants in time and explain medication to them. Compliance of subjects will be evaluated by the below formula. Subjects are asked to return remaining drugs.

Compliance(%) = [(distributed drugs-remained drugs)/distributed drugs] ×100

### Statistical analyses

#### Efficacy analysis

All analyses in this study will be based on both intention-to-treat (ITT) and per protocol (PP) method. The ITT method will include all randomized subjects who made at least one next visit. The participants who have completed the 6-week treatment without major protocol violations and kept compliance rate over 80% will be analyzed by the pp (per protocol) method. The continuous variables will be summarized as mean±SD (standard deviation) and the categorical variables will be described in frequency and percent. For the primary and secondary outcome variables, the mean differences between before (visit 2; baseline) and after the treatment (visit 4) values will be compared using the paired t-test or Wilcoxon test, in each group. The baseline characteristics will be compared by either the Student t-test for the continuous variables or χ^2^-test for the categorical data (alternatively, Fisher's exact test will be used if the expected value is less than 5 or McNemar's test for non-normal distribution data). The efficacy of PURIAM110 will be assessed by using the independent t-test (when normality assumption is satisfied, alternatively Mann-Whitney test will be used). All analyses will be based on a two-sided test at a 5% significance level. With values of p < 0.05 will be considered statistically significant. SPSS for windows version 12.0 (SPSS Inc., Chicago, Illinois) will be used for analyses. For missing values, 'Last Observation Carried Forward (LOCF)' method will be applied.

#### Safety analysis

In the safety analysis, it will include the subjects who were both randomized and treated with the investigational drug at least once. There will be stratification by the institution and the adverse event symptom.

### Adverse event (AE) reporting

All AE must be observed and documented in the CRF AE form. When AE happens, researchers must report it to both IRB and regulatory authorities within 24 hours.

### Data collection

After filling out the CRF, data collection will be performed according to the standard operating procedures (SOPs), by the trained clinical research associates (CRAs).

### Data management and monitoring

For data accuracy and trial quality, monitoring and data management will be carried out by the authorized contract research organization (CRO), Center for Clinical Research and Genomics (CCRG), Seoul, Korea.

### Ethical approvals

The study has been accepted by the Institutional Review Board (IRB) of two hospitals (Institutional Review Board of the Kyung Hee University Oriental Medical Center approved on the 21th of November 2006 (ref: KOMC IRB 2006-14), and Institutional Review Board of the Kyung Won Gil Oriental Medical Hospital approved on the 25th of October 2006(ref: 06-101)). Informed consent form will be provided to each individual prior to the enrolment. The research will be performed in compliance with the Helsinki Declaration and the Good Clinical Practice (GCP) Guidelines.

## Discussion

In this paper, we have suggested the clinical trial design of a pre-diabetes treating dietary supplement. This pilot study will be the groundwork for the larger scale RCT. To draw confirmative conclusion about the therapeutic efficacy and safety of pre-diabetes supplements, a full-scale RCT has to be done. To proceed with the trial, two criteria must be decided through this study. First, the adequate sample size has to be calculated. As there were no published studies with similar outcomes, we had difficulties in obtaining the mean and variation estimates for the study. Therefore, we designed a pilot trial to figure out the mean and standard deviation. Besides, this study result will also provide a magnitude of the clinically significant treatment effect. With these estimates, we will be able to compute the exact sample size of the larger multicenter study. Second, the primary endpoint has to be fixed. The current preclinical study results suggest that bioflavonoids, hesperidin, and naringin, which are contained in mulberry leaves and bitter oranges, prevent not only hyperglycemia but hyperlipidemia in type-2 diabetic animals [17,18,21,22,29,30]. Based on these consequences, we decided to include lipid profile (total cholesterol, triglyceride, LDL cholesterol) indexes, in addition to the glucose related parameters (HbA1c, fructosamine, fasting glucose, 2-h OGTT glucose, fasting insulin). Among the glucose and the lipid related parameters, we will decide the primary outcome for a large scale trial according to the result of this study. Furthermore, as anti-diabetic agents show ethnic differences, PURIAM110 has to be verified by more various groups.

## List of abbreviations used

KNHNS: Korean National Health and Nutrition Survey; IFG: impaired fasting glucose; IGT: impaired glucose tolerance; KFDA: Korea Food & Drug Administration; RCT: Randomized controlled trial; LDL: low density lipoprotein; OGTT: oral glucose tolerance test; WC: waist circumference; HC: hip circumference; VAS: visual analogue scale; SOP: standard operating procedure; QSCC II: Questionnaire for the Sasang Constitution Classification II; ITT: intention-to-treat; PP: per protocol; SD: standard deviation; LOCF: Last Observation Carried Forward; CRF: case report form; AE: adverse event; CRA: clinical research associates; CRO: contract research organization; IRB: Institutional Review Board; GCP: Good Clinical Practice

## Competing interests

The authors declare that they have no competing interests.

## Authors' contributions

SKG substantially contributed to the general idea and design of the study. SJP, BHC, YCS, HYK, JSP took part in designing the protocol. SJP, BHC, YCS planned data analysis and SJP drafted the manuscript. All authors have read and consented to the manuscript.

## References

[B1] Hsueh WA Orloski L Wyne K Prediabetes: the importance of early identification and intervention Postgrad Med 2010 122 129 143 10.3810/pgm.2010.07.2180 20675976

[B2] Gillman MW Predicting prediabetes and diabetes: can we do it? Is it worth it? Arch Pediatr Adolesc Med 2010 164 198 199 10.1001/archpediatrics.2009.270 20124151 PMC4350017

[B3] Alberti KG Zimmet PZ Definition, diagnosis and classification of diabetes mellitus and its complications. Part 1: diagnosis and classification of diabetes mellitus provisional report of a WHO consultation Diabet Med 1998 15 539 553 10.1002/(SICI)1096-9136(199807)15:7<539::AID-DIA668>3.0.CO;2-S 9686693

[B4] Kim SM Lee JS Lee J Na JK Han JH Yoon DK Baik SH Choi DS Choi KM Prevalence of diabetes and impaired fasting glucose in Korea: Korean National Health and Nutrition Survey 2001 Diabetes Care 2006 29 226 231 10.2337/diacare.29.02.06.dc05-0481 16443864

[B5] Report of the expert committee on the diagnosis and classification of diabetes mellitus Diabetes Care 2003 26 Suppl 1 S5 20 12502614 10.2337/diacare.26.2007.s5

[B6] Annual report on the cause of death statistics Book Annual report on the cause of death statistics (Editor ed.^eds.) 2008 City: Statistics Korea

[B7] Harris MI Flegal KM Cowie CC Eberhardt MS Goldstein DE Little RR Wiedmeyer HM Byrd-Holt DD Prevalence of diabetes, impaired fasting glucose, and impaired glucose tolerance in U.S. adults. The Third National Health and Nutrition Examination Survey, 1988-1994 Diabetes Care 1998 21 518 524 10.2337/diacare.21.4.518 9571335

[B8] Grant SJ Bensoussan A Chang D Kiat H Klupp NL Liu JP Li X Chinese herbal medicines for people with impaired glucose tolerance or impaired fasting blood glucose Cochrane Database Syst Rev 2009 CD006690 19821382 10.1002/14651858.CD006690.pub2 PMC3191296

[B9] Cho NH The epidemiology of diabetes in Korea: from the economics to genetics Korean Diabetes J 2010 34 10 15 10.4093/kdj.2010.34.1.10 20532014 PMC2879903

[B10] Kim CH Early insulin secretory dysfunction in korean prediabetic subjects: should we change the criteria for "prediabetes?" Korean Diabetes J 2010 34 154 156 10.4093/kdj.2010.34.3.154 20617075 PMC2898928

[B11] Gavi S Hensley J Diagnosis and management of type 2 diabetes in adults: a review of the ICSI guideline Geriatrics 2009 64 12 17 29 19572762

[B12] Li WL Zheng HC Bukuru J De Kimpe N Natural medicines used in the traditional Chinese medical system for therapy of diabetes mellitus J Ethnopharmacol 2004 92 1 21 10.1016/j.jep.2003.12.031 15099842

[B13] Yeh GY Eisenberg DM Kaptchuk TJ Phillips RS Systematic review of herbs and dietary supplements for glycemic control in diabetes Diabetes Care 2003 26 1277 1294 10.2337/diacare.26.4.1277 12663610

[B14] Shapiro K Gong WC Natural Products Used for Diabetes J Am Pharm Assoc 2002 42 217 226 10.1331/108658002763508515 11926665

[B15] Malviya N Jain S Malviya S Antidiabetic potential of medicinal plants Acta Pol Pharm 2010 67 113 118 20369787

[B16] Hunt LM Arar NH Akana LL Herbs, Prayer, and Insulin Use of Medical and Alternative Treatments by a Group of Mexican American Diabetes Patients J Fam Pract 2000 49 216 223 10735480

[B17] Jung UJ Lee MK Jeong KS Choi MS The hypoglycemic effects of hesperidin and naringin are partly mediated by hepatic glucose-regulating enzymes in C57BL/KsJ-db/db mice J Nutr 2004 134 2499 2503 15465737 10.1093/jn/134.10.2499

[B18] Jung UJ Lee MK Park YB Kang MA Choi MS Effect of citrus flavonoids on lipid metabolism and glucose-regulating enzyme mRNA levels in type-2 diabetic mice Int J Biochem Cell Biol 2006 38 1134 1145 10.1016/j.biocel.2005.12.002 16427799

[B19] Kim H Herbal pharmacology 2001 Seoul: Jipmoondang

[B20] Orange Fruit http://www.fzrm.com/plantextracts/Orange%20_Fruit_extract.htm

[B21] Andallu B Suryakantham V Lakshmi Srikanthi B Reddy GK Effect of mulberry (Morus indica L.) therapy on plasma and erythrocyte membrane lipids in patients with type 2 diabetes Clin Chim Acta 2001 314 47 53 10.1016/S0009-8981(01)00632-5 11718678

[B22] Andallu B Varadacharyulu N Antioxidant role of mulberry (Morus indica L. cv. Anantha) leaves in streptozotocin-diabetic rats Clin Chim Acta 2003 338 3 10 10.1016/S0009-8981(03)00322-X 14637259

[B23] Muberry Leaf http://www.fzrm.com/plantextracts/Muberry_Leaf_extract.htm

[B24] Traditional medicine college textbook compilation committee of herbology Herbology 2007 Seoul: Yeongnimsa

[B25] Wood DM Athwal S Panahloo A The advantages and disadvantages of a 'herbal' medicine in a patient with diabetes mellitus: a case report Diabet Med 2004 21 625 627 10.1111/j.1464-5491.2004.01202.x 15154951

[B26] Diagnosis and classification of diabetes mellitus Diabetes Care 2010 33 Suppl 1 S62 69 20042775 10.2337/dc10-S062 PMC2797383

[B27] Kim JI Kim JC Kang MJ Lee MS Kim JJ Cha IJ Effects of pinitol isolated from soybeans on glycaemic control and cardiovascular risk factors in Korean patients with type II diabetes mellitus: a randomized controlled study Eur J Clin Nutr 2005 59 456 458 10.1038/sj.ejcn.1602081 15536472

[B28] World Health Organization WHO International Standard Terminologies on Traditional Medicine in the Western Pacific Region. Geneva 2007

[B29] Liu JC Chan P Hsu FL Chen YJ Hsieh MH Lo MY Lin JY The in vitro inhibitory effects of crude extracts of traditional Chinese herbs on 3-hydroxy-3-methylglutaryl-coenzyme A reductase on Vero cells Am J Chin Med 2002 30 629 636 10.1142/S0192415X02000454 12568290

[B30] Seo HJ Jeong KS Lee MK Park YB Jung UJ Kim HJ Choi MS Role of naringin supplement in regulation of lipid and ethanol metabolism in rats Life Sci 2003 73 933 946 10.1016/S0024-3205(03)00358-8 12798418

